# The cardiothoracic ratio and all-cause and cardiovascular disease mortality in patients undergoing maintenance hemodialysis: results of the MBD-5D study

**DOI:** 10.1007/s10157-017-1380-2

**Published:** 2017-05-15

**Authors:** Hiroaki Ogata, Junji Kumasawa, Shingo Fukuma, Masahide Mizobuchi, Eriko Kinugasa, Masafumi Fukagawa, Shunichi Fukuhara, Tadao Akizawa

**Affiliations:** 1 Department of Internal Medicine, Showa University Northern Yokohama Hospital, Chigsaki-chuo 35-1, Tsuzuki, Yokohama, Kanagawa 224-8503 Japan; 2 Department of Healthcare Epidemiology, Graduate School of Medicine and Public Health, Kyoto University, Kyoto, Japan; 3 Department of Critical Care Medicine, Sakai City Medical Center, Osaka, Japan; 4 Institute for Health Outcomes and Process Evaluation Research (iHope International), Kyoto, Japan; 5 Center for Innovative Research for Communities and Clinical Excellence (CIRC2LE), Fukushima Medical University, Fukushima, Japan; 6 Division of Nephrology, Department of Medicine, Showa University School of Medicine, Tokyo, Japan; 7 Division of Nephrology, Endocrinology and Metabolism, Tokai University School of Medicine, Kanagawa, Japan

**Keywords:** Cardiothoracic ratio, Cardiovascular disease, CKD-MBD, MBD-5D study, Hemodialysis

## Abstract

**Background:**

The cardiothoracic ratio (CTR) is a non-invasive left ventricular hypertrophy index. However, whether CTR associates with cardiovascular disease (CVD) and mortality in hemodialysis (HD) populations is unclear.

**Methods:**

Using a Mineral and Bone disorder Outcomes Study for Japanese CKD Stage 5D Patients (MBD-5D Study) subcohort, 2266 prevalent HD patients (age 62.8 years, female 38.0%, HD duration 9.4 years) with secondary hyperparathyroidism (SHPT) whose baseline CTR had been recorded were selected. We evaluated associations between CTR and all-cause death, CVD death, or composite events in HD patients.

**Results:**

CTR was associated significantly with various background and laboratory characteristics. All-cause death, CVD-related death, and composite events increased across the CTR quartiles (Q). Adjusted hazard risk (HR) for all-cause death was 1.4 (95% confidential interval, 0.9–2.1) in Q2, 1.9 (1.3–2.9) in Q3, and 2.6 (1.7–4.0) in Q4, respectively (Q1 as a reference). The corresponding adjusted HR for CVD-related death was 1.8 (0.8–4.2), 3.1 (1.4–6.8), and 3.5 (1.6–7.9), and that for composite outcome was 1.2 (1.0–1.6), 1.7 (1.3–2.2), and 1.8 (1.5–2.3), respectively. Exploratory analysis revealed that there were relationships between CTR and age, sex, body mass index, comorbidity of CVD, dialysis duration, dialysate calcium level and intact parathyroid hormone, phosphorus, hemoglobin, and usage of phosphate binder.

**Conclusion:**

CTR correlated with all-cause death, CVD death, and composite events in HD patients with SHPT.

**Electronic supplementary material:**

The online version of this article (doi:10.1007/s10157-017-1380-2) contains supplementary material, which is available to authorized users.

## Introduction

Left ventricular hypertrophy (LVH) is a significant predictor of cardiovascular morbidity and mortality in patients undergoing long-term hemodialysis (HD) therapy [1–7]. Many factors in the uremic milieu, including hypertension, volume overloading, anemia, chronic kidney disease-mineral and bone disorder (CKD-MBD), the oxidative state, and inflammation, have been implicated in LVH pathogenesis [6–8]. The cardiothoracic ratio (CTR) is a readily available and non-invasive tool with which to assess the volume status and cardiomegaly. In addition, the CTR was found to correlate independently with LVH and target organ damage in hypertensive patients [9]. The CTR is likely to be a predictor all-cause and cardiovascular disease (CVD) mortality in patients with mild-to-moderate heart failure [9–11] or those undergoing coronary angiography [12]. However, it remains unclear that a higher CTR is associated with increased mortality among a hemodialysis-treated population [13–16]. This study was designed to evaluate whether CTR could predict all-cause death, CVD death, and the combined outcome of all-cause death and CVD-relate hospitalization in long-term HD patients, using data from the Mineral and Bone Disorder Outcomes Study for Japanese CKD Stage 5D patients (MBD-5D study) [17–21].

## Methods

### Study design and patients

The MBD-5D study was a 3-year prospective observational study involving hemodialysis patients with secondary hyperparathyroidism. The entire study cohort comprised 8229 enrolled patients, from whom a subcohort comprising 3276 patients was selected. The study involved relatively large dialysis facilities (each catering for >100 patients) in Japan, and patients were eligible for inclusion if they had consistently received hemodialysis for more than 3 months at the participating facilities as of January 1st, 2008, and if they met at least one of the following conditions: an intact parathyroid hormone (iPTH) level of >180 pg/ml or receiving treatment with intravenous vitamin D receptor activators (VDRAs; calcitriol or maxacalcitol) or oral VDRA (falecalcitriol). Patients were excluded if they had undergone hemodialysis for less than 3 months at the time of evaluation for inclusion.

Details of the study design were reported previously [17, 18]. In this study, all patients whose baseline cardiothoracic ratio (CTR) had been recorded were enrolled.

### Exposure, outcomes, and covariates

The CTR was measured by chest radiography in the postero-anterior view, while the patients were standing. The CTR was calculated by dividing the maximal horizontal heart breadth by the horizontal inner rib cage breadth. To confirm the accuracy of the measurements, a vertical line was drawn on each radiograph through the spinal midpoint from the sternum to the diaphragm. The maximum transverse cardiac diameter was obtained by adding the widest distance from the right heart border to the midline and the distance from the left cardiac border to the midline. At each facility, the CTR was measured at the start of the study and every 12 months throughout the study. Although the timing of CTR measurements was not clearly determined in the MBD-5D study, standard chest X-ray is performed immediately before the first HD session in a week in most Japanese dialysis facilities [22–24].

Patients were classified into four groups based on the baseline CTR quartiles: ≤46.8, 46.8, <−50, 50, <−53.6, and >53.6%. As the CTR values changed during the study period, the CTR groups were expressed as time-varying covariates. It was permissible for patients to be placed in only one group at a time, although group placement during the study period changed according to the CTR.

The main outcome measure was all-cause death. The secondary outcome measures were cardiovascular disease (CVD)-related death and the combined outcome of all-cause death and CVD-related hospitalization.

We also collected covariate demographic data (age, sex, BMI, smoking status, comorbidity of CVD and diabetes mellitus, and dialysis duration), baseline laboratory data (levels of iPTH, phosphorus, calcium, hemoglobin, albumin, and C-reactive protein), and covariate data related to dialysis (single-pool Kt/V and dialysate calcium level) and other medications (phosphate binder, vitamin D receptor activator, ACE inhibitor, and beta blocker) at the start of the study.

### Statistical methods

Continuous variables were expressed as the mean ± standard deviation, whereas categorical variables were expressed as a proportion. Continuous variables were compared among the groups using the analysis of variance (ANOVA). Categorical variables were compared using the Chi-squared test. We analyzed the adjusted hazard ratios (HRs) for all-cause death, CVD-related death, and the combined outcome of all-cause death and CVD-related hospitalization using the Cox proportional hazards model with the CTR level as a time-varying covariate. In the Cox proportional hazards model, missing CTR values other than the baseline value were replaced by the last observed CTR value. In all analyses, the CTR ≤ 46.8% group was used as a reference. All models were adjusted for all the covariates listed in Table 1.

**Table 1:** Patient baseline characteristics

Variable	ALL	Q1	Q2	Q3	Q4	*P*
		CTR ≤ 46.8%	CTR 46.8% <−50%	CTR 50% <−53.6%	CTR > 53.6%	
	(*n* = 2266)	(*n* = 574)	(*n* = 596)	(*n* = 531)	(*n* = 565)	
Age (year)	62.8 (12.9)	58.3 (13.1)	61.6 (12.1)	63.7 (12.1)	67.6 (12.3)	<0.001
Female (%)	38.0	27.0	29.5	41.1	55.0	<0.001
Dialysis duration (year)	9.4 (8.2)	9.0 (8.0)	9.2 (7.6)	9.9 (8.3)	9.8 (8.8)	0.171
CVD (%)	60.6	50.4	58.1	61.8	72.6	<0.001
DM (%)	34.6	34.7	35.7	34.3	33.6	0.896
Smoking	10.7	12.9	10.7	10.0	9.2	0.213
BMI (kg/m^2^)	21.3 (3.6)	21.4 (3.0)	21.7 (3.3)	21.3 (4.0)	20.8 (3.8)	<0.001
Kt/V	1.4 (0.3)	1.4 (0.3)	1.4 (0.3)	1.4 (0.3)	1.4 (0.3)	<0.001
Ca (mg/dL)	9.3 (0.9)	9.3 (0.8)	9.3 (0.9)	9.3 (0.9)	9.4 (0.9)	0.576
P (mg/dL)	5.5 (1.4)	5.5 (1.4)	5.6 (1.4)	5.5 (1.3)	5.5 (1.4)	0.824
iPTH (pg/mL)	314.5 (244.0)	280.1 (172.4)	321.9 (228.9)	317.6 (246.3)	338.6 (308.0)	<0.001
Hb (g/dL)	10.5 (1.2)	10.7 (1.2)	10.6 (1.2)	10.4 (1.1)	10.2 (1.2)	<0.001
ALB (mg/dL)	3.7 (0.4)	3.8 (0.4)	3.8 (0.3)	3.7 (0.4)	3.6 (0.4)	<0.001
CRP (mg/dL)	0.5 (1.4)	0.5 (1.6)	0.4 (1.5)	0.3 (0.7)	0.6 (1.5)	0.030
VDRA use (%)	76.0	78.8	76.3	75.1	73.8	0.249
Phosphate binder use (%)	83.4	82.6	85.4	83.1	82.5	0.493
ACE inhibitor use (%)	7.7	5.9	8.1	10.4	6.7	0.034
ARB use (%)	36.3	39.6	38.9	34.7	31.7	0.017
Beta blocker use (%)	6.1	6.3	8.4	4.9	4.8	0.037
Dialysate Ca (mEq/L)	2.7 (0.2)	2.7 (0.2)	2.7 (0.2)	2.7 (0.2)	2.7 (0.3)	0.113

*CVD* cardiovascular-related disease, *DM* diabetes mellitus, *BMI* body mass index, *Ca* calcium, *P* phosphorus, *ACE inhibitor* angiotensin converting enzyme inhibitor, *ARB* angiotensin receptor blocker

We also performed the same analysis stratified by the presence of DM and age (<64 year, >65 year). We performed a sensitivity analysis using all the covariates as time-varying covariates. In this analysis, any missing covariates were replaced by the last observed values of those variables.

As an exploratory analysis, we performed multivariable regression analysis to investigate the relationship between CTR and covariates listed above.

Differences were considered statistically significant at a two-sided *p* value of ≤0.05. All statistical calculations were performed using STATA version 13 (STATA, College Station, TX).

## Results

### Patient characteristics

**Fig. 1:**
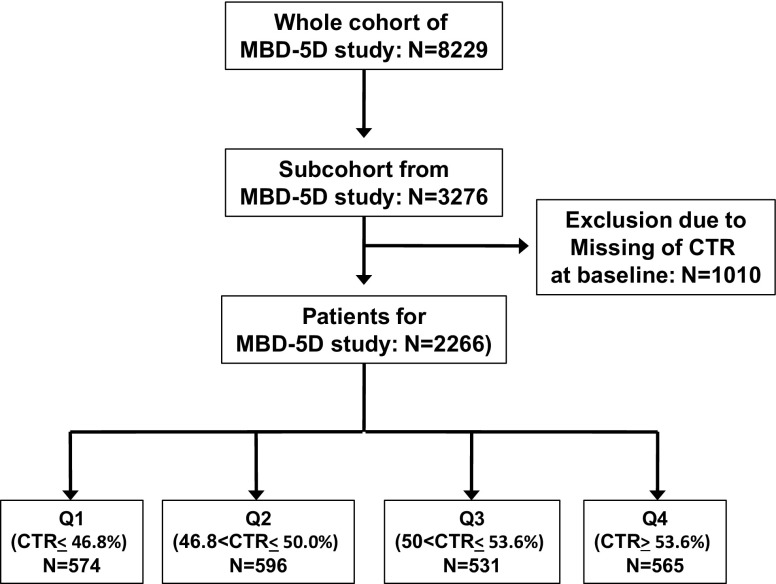
Patient flow chart. Patients for the study subcohort were randomly selected from whole MBD-5D study cohort. Of the 3276 patients in the subcohort, 1010 were excluded due to missing CTR values at the baseline. Based on the CTR value at the baseline, 574, 596, 531, and 565 patients were placed into the CTR ≤46.8, 46.8, <−50, 50, <−53.6, and >53.6% groups, respectively. *MBD-5D* mineral and bone disorder outcomes study for Japanese chronic kidney disease stage 5D patients. *CTR* cardiothoracic ratio

In this study, 2266 patients were analyzed. In terms of the baseline CTR values, 574 patients were placed in the ≤46.8% group, 596 in the 46.8, <−50% group, 531 in the 50, <−53.6% group, and 565 in the >53.6% group. Figure 1 shows a flow chart of the patients, and Table 1 shows the baseline clinical characteristics of the study population according to the CTR values at the start of the study. Table 1 shows the baseline clinical characteristics of the study population according to the CTR values at the start of the study. With regard to patient characteristics, the mean age was 62.8 years, the proportion of females was 38%, and mean duration of dialysis was 9.4 years. Age, gender, CVD morbidities, body mass index, Kt/V, intact PTH, Hb, albumin, C-reactive protein, renin-angiotensin system inhibitors use, and beta blockers use were significantly different among patients with each of the CTR quartiles.

### Association between CTR levels and mortality or composite outcome

Of the 2266 patients, 404 (17.8%) died due to any cause, 151 (6.7%) died due to CVD-related events, and 835 composite events occurred during the study period. Table 2 shows the number of events that occurred and the proportion of events that occurred in the various CTR value groups.

**Table 2:** Event occurrence during the study period

	Q1		Q2		Q3		Q4		Total	
	CTR ≤ 46.8%		CTR 46.8% <−50%		CTR 50% <−53.6%		CTR > 53.6%			
	(*n* = 574)		(*n* = 596)		(*n* = 531)		(*n* = 565)		(*n* = 2266)	
	Proportion	No. of events	Proportion	No. of events	Proportion	No. of events	Proportion	No. of events	Proportion	No. of events
All-cause death	8.0	46	12.1	72	19.6	104	32.2	182	17.8	404
CVD-related death	2.1	12	4.4	26	8.1	43	12.4	70	6.7	151
Composite events	23.2	133	28.9	172	41.6	221	54.7	309	36.8	835

*Composite events* combined outcome of all-cause death and CVD-related hospitalization

Table 3a, b shows the unadjusted and adjusted HRs calculated using the Cox proportional hazards model with time-varying covariates. All-cause death, CVD-related death, and composite events increased as the CTR value increased.

**Table 3:** Results of Cox proportional hazards model with time-varying covariates

	Q2	Q3	Q4	Number of patients
	CTR 46.8% <−50%	CTR 50% <−53.6%	CTR > 53.6%	
	HR^a^ (95% CI)			
(a) Unadjusted				
All-cause death	1.4 (1.0–2.1)	2.4 (1.7–3.5)	4.0 (2.9–5.7)	2266
CVD-related death	2.0 (1.0–3.9)	3.8 (2.0–7.5)	5.7 (3.1–10.8)	2266
Composite events	1.2 (1.0–1.5)	1.8 (1.4–2.3)	2.4 (1.9–2.9)	2266
(b) Adjusted				
All-cause death	1.4 (0.9–2.1)	1.9 (1.3–2.9)	2.6 (1.7–4.0)	1954
CVD-related death	1.8 (0.8–4.2)	3.1 (1.4–6.8)	3.5 (1.6–7.9)	1954
Composite events	1.2 (1.0–1.6)	1.7 (1.3–2.2)	1.8 (1.5–2.3)	1954

In these analyses, the CTR ≤ 46.8% group (Q1) was used as a reference

*Composite events* combined outcome of all-cause death and CVD-related hospitalization

^a^Unadjusted hazard ratio. CTR was treated as a time-varying covariate

^b^Adjusted for age, sex, BMI, smoking status, comorbidity of CVD, diabetes mellitus, dialysis duration, levels of iPTH, phosphorus, calcium, hemoglobin, albumin and C-reactive protein, Kt/V, dialysate calcium level, phosphate binder, vitamin D receptor activator, ACE inhibitor, and beta blocker

**Fig. 2:**
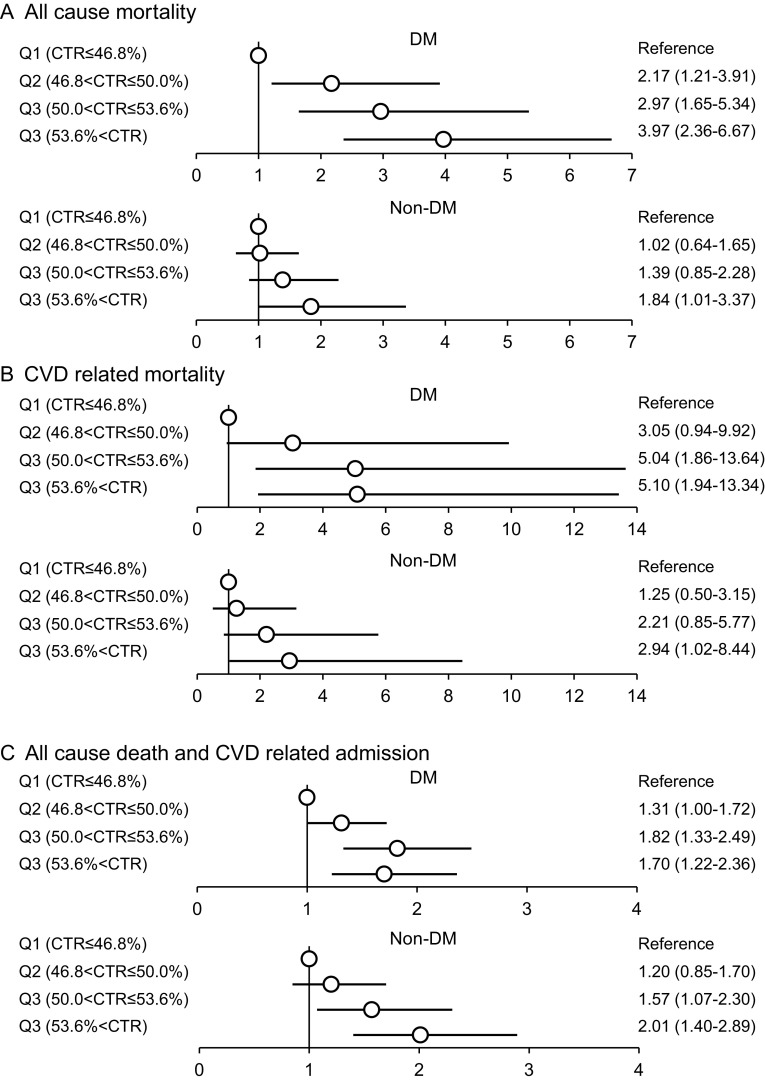
Subgroup analysis stratified by the presence of diabetes mellitus (DM). *Vertical line* indicates the reference level. The adjusted HRs are shown with point estimates and 95% confidence intervals. HRs were adjusted by age, sex, BMI, smoking status, comorbidity of CVD, diabetes mellitus, dialysis duration, levels of iPTH, phosphorus, calcium, hemoglobin, albumin and C-reactive protein, Kt/V, dialysate calcium level, phosphate binder, vitamin D receptor activator, ACE inhibitor, and beta blocker

**Fig. 3:**
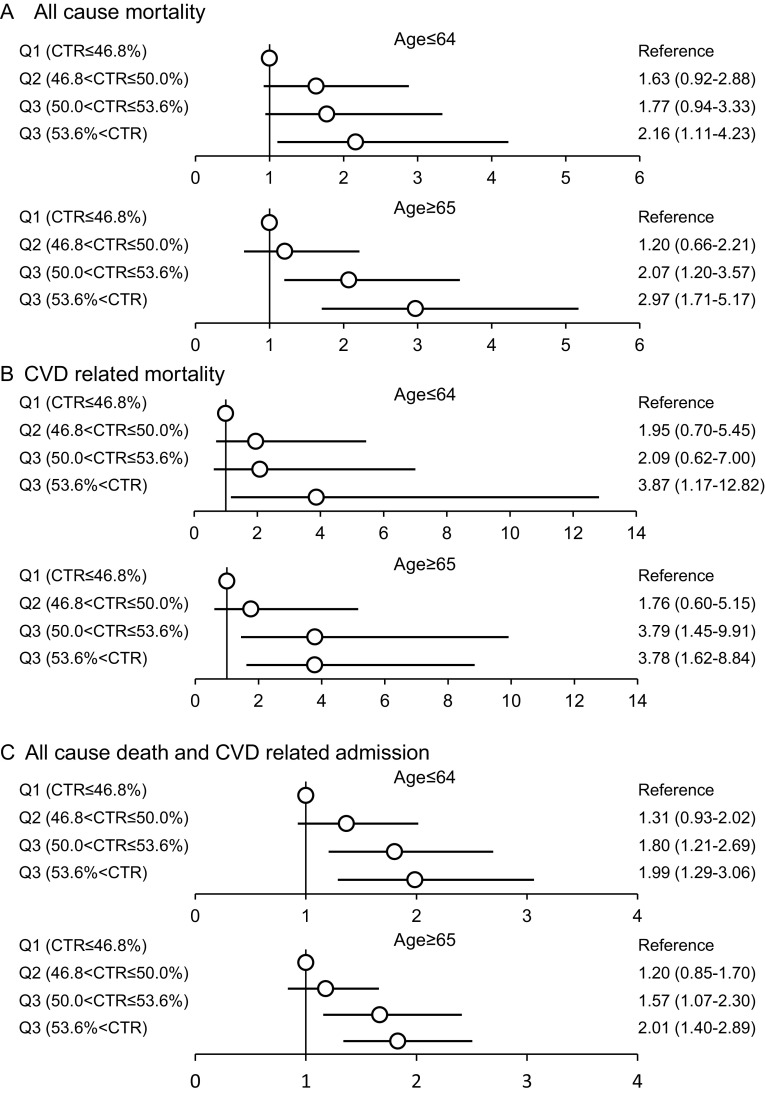
Subgroup analysis stratified by age (< 64 year, >65 year). Vertical line indicates the reference level. The adjusted HRs are shown with point estimates and 95% confidence intervals. HRs were adjusted by age, sex, BMI, smoking status, comorbidity of CVD, diabetes mellitus, dialysis duration, levels of iPTH, phosphorus, calcium, hemoglobin, albumin and C-reactive protein, Kt/V, dialysate calcium level, phosphate binder, vitamin D receptor activator, ACE inhibitor, and beta blocker

Figures 2 and 3 show the subgroup analysis stratified by the presence of DM and age (<64, >65 years). These analyses demonstrated the same pattern of associations with CTR and mortality.

### Sensitivity analysis

Sensitivity analysis using all the covariates as time-varying covariates yielded results similar to those of the main analysis (Supplement).

### Exploratory analysis investigating the relationship between CTR and covariates

Multivariable regression analysis showed that there were relationships between CTR and age, sex, BMI, comorbidity of CVD, dialysis duration, dialysate calcium level and level of iPTH, phosphorus, hemoglobin, and usage of phosphate binder (Table 4).

**Table 4:** Exploratory analysis on determinants of CTR

Variable	Unit	*β*coefficients	95% CI	*P* value
Age	per 1 year	0.093	0.070 to 0.117	<0.001
Sex, female	vs Male	1.664	(1.040 to 2.287)	<0.001
Diabetes mellitus comorbidity	vs No	−0.215	(−0.748 to 0.318)	0.424
Cardiovascular-related disease comorbidity	vs No	1.176	(0.596 to 1.756)	<0.001
Diabetes mellitus comorbidity	vs No	−0.215	(−0.748 to 0.318)	0.424
Smoking	vs No	0.736	(−0.145 to 1.618)	0.100
Body mass index	per 1 kg/m^2^	0.224	(0.079 to 0.369)	0.003
Urea clearance time/volume	per 0.1	−0.602	(−1.872 to 0.667)	0.348
Dry weight	per 1 kg	−0.096	(− 0.145 to − 0.046)	<0.001
Calcium	per 1 mg/dL	−0.409	(−0.341 to 0.259)	0.786
Phosphorus	per 1 mg/dL	0.211	(0.025 to 0.398)	0.027
Intact parathyroid hormone	per 100 pg/mL	0.002	(0.001 to 0.003)	<0.001
Hemoglobin	per 1 g/dL	−0.300	(− 0.528 to − 0.073)	0.010
Albumin	per 1 g/dL	−0.342	(−1.013 to 0.329)	0.313
C-reactive protein	per 1 mg/dL	0.046	(−0.117 to 0.209)	0.576
Vitamin D receptor activator	vs No	0.312	(−0.525 to 0.588)	0.911
Phosphate binder	vs No	0.746	(0.072 to 1.420)	0.031
Angiotensin converting enzyme inhibitor	vs No	0.145	(−0.729 to 1.019)	0.741
Beta blocker	vs No	−0.068	(−1.317 to 1.181)	0.914

## Discussion

CVD is more prevalent among HD patients and is the main cause of death [25, 26]. Epidemiological studies have demonstrated that the CTR was a significant predictor of both all-cause and CVD-related mortality in patients regardless of undergoing dialysis therapy [10–16]. However, this study is a large scale, and is the first study to investigate the relationship between CTR measurement and clinical outcomes using a time-average Cox regression model in ESKD patients, focusing on CTR measurements as a time-dependent covariate. Thus, the results of this study provide strong evidence that CTR corrected with mortality among dialysis population. The CTR measurement timing was thus more prolonged when compared with the timing used in other studies. In this study, we used a time-dependent Cox regression model to perform a detailed analysis of the associations between the CTR and the outcomes in a large subpopulation from the study patients of MBD-5D study [17–21], because the CTR measurements fluctuated in our large cohort study. After adjusting for various factors, higher CTR measurements were significantly associated with higher risks of all-cause and CVD mortality in this cohort. This study demonstrates that HRs for all-cause death, CVD-related death, and composite events (all-cause death and CVD-related hospitalization) increase across the CTR quartiles. In the subgroup analysis stratified by the presence of diabetes mellitus and age (≤64, ≥65 year), there was seen the same pattern of association between CTR and mortality or the composite outcome. In an exploratory assessment using multivariable regression analysis, age, gender, body mass index, dry weight, CVD comorbidities, Hb, P, iPTH, and P-binder use were significantly associated with CTR.

The CTR is an easy, reliable, and inexpensive tool for evaluating LVH and volume status without using specific equipment or technical modalities, such as ultrasonic echocardiography or magnetic resonance imaging (MRI). Furthermore, the CTR was found to independently correlate with the LV mass (LVM) and target organ damage [9]. The previous studies have shown that the CTR predicted systolic ventricular dysfunction and sudden death in patients with chronic heart failure [11, 27]. LVH is an independent and strong predictor of all-cause and CVD mortality and CVD events in dialysis patients [1–7]. Many pathologic factors, including hypertension, volume overloading, local or systemic renin-angiotensin activation, sympathetic hyperactivity, inflammation, and oxidative stress, contribute to LVH in HD patients. In addition to classical factors, CKD-MBD also plays a crucial role in the LVH development [6, 7, 28–30]. Excess PTH and P overloading can induce cardiovascular remodeling in uremic animal models [31, 32]. Cardiac MRI, the most frequently used tool for cardiac remodeling assessments, was used to demonstrate that Ca × P products as well as the end-diastolic LV volume and pre-dialysis blood pressure levels could predict the LVM index in HD patients [33]. Recently, Yamamoto et al. reported an association between higher dietary P consumption and greater LVM in a community-based multiethnic population without clinical CVD [34]. P overloading stimulates an increase in the serum fibroblast growth factor-23 levels and a decrease in 1,25-dihydroxyvitamin D production. These hormonal changes, which serve to maintain P homeostasis, have been implicated in cardiovascular remodeling in both experimental and observational studies 35–37. Interestingly, this study found that higher serum P levels and P-binder usage were associated with a higher CTR, which likely indicates increased LVH, in patients who were undergoing HD with SHPT. No previous studies have reported a significant association between P levels or P-binder usage and CTR values. Beyond doubt, the observational study never establishes any cause-and-effect conclusion. Further studies need to elucidate effect of P management on CTR measurements in CKD patients.

Agarwal demonstrated that hypervolemia, which is detected via relative plasma volume monitoring, could predict higher mortality among HD patients [38]. The CTR could significantly predict the volume status in HD patients. An observational study revealed that CTR-guided ultrafiltration management led to improvements in hypertension and cardiomegaly [39] and that a strict volume control strategy improved survival and reduced the CTR values even in patients undergoing the conventional HD treatment [40]. Therefore, the CTR is a useful volume status indicator in HD patients and can be modified through aggressive volume control management.

This study has several strengths and limitations with respect to interpretations of the results. First, given its observational nature, this study could not establish an obvious causal relationship between the CTR and clinical outcomes. Second, the study population was younger and had longer vintage of HD compared with general Japanese dialysis patients, although they were recruited from a large, multicenter cohort study in Japan (MBD-5D study). In addition, only the patients with SHPT were included in this study. Therefore, they might not have representability of general Japanese dialysis patients. Third, although it is very important whether the addition of CTR measurement to predictive model with patients’ characteristics at baseline could be more predictive of mortality, time-dependent Cox regression model, which is used in this study, is unsuitable for validating it. Bohn et al. reported that CTR was independently associated with mortality in retrospective cohort study of 824 HD patients, but that CTR did not significantly improve predictive power for mortality when CTR was added to a base clinical model [16]. Hyperphosphatemia and P-binder usage were significant CTR determinants in this study population and were representative of CKD-MBD and SHPT. These results might also be specific to patients with established SHPT. However, a large number of patients with a very wide range of serum iPTH levels were carefully evaluated in this study; therefore, we believe that this limitation is negligible. Finally, arrhythmia (particularly atrial fibrillation) and heart valvular diseases, which are more prevalent in hemodialysis patients, can also contribute to higher CTR values. However, those factors were not assessed in detail in this study. Given these limitations, these results should be interpreted and generalized with caution.

This study has demonstrated that the CTR, which is associated with various clinical parameters, is significantly predictive of all-cause and CVD mortality, and composite outcomes in patients receiving HD. These results suggest that efforts of reduced CTR or prevention of its enlargement, including an aggressive volume control strategy, optimal anemia, and CKD-MBD management, could potentially reduce the CVD morbidity and mortality rates in HD patients.

### Electronic supplementary material

Below is the link to the electronic supplementary material.

Supplementary material 1 (DOCX 143 KB)

## Appendix

We would like to thank the following MBD-5D study investigators who participated in this study: Nobuo Hashimoto (H.N.MEDIC), Mari Ishida (Kitasaito Hospital), Toshiyuki Date (Date Clinic), Kiyotaka Yabuki (Yabuki Hospital), Hideki Tanida (Tendo Onsen Yabuki Clinic), Fumitoshi Yamauchi (San-ai Hospital), Mikihiko Fujishima (Yahaba Clinic), Tomohito Matsunaga (Eijinkai Hospital), Jun Urae (Ishinomaki Clinic), Hiroshi Kawaguchi (Iwaki Urological Hospital), Ikuo Takahashi (Kisen Hospital), Yoshiko Tanaka (Shinjuku Ishikawa Clinic), Hideo Kobayashi (Suda Clinic), Maki Takahashi (Suda Naika Clinic), Tatsuya Nonaka (Seishokai Memorial Hospital), Hideto Emoto (Tokai Hospital), Kyosuke Nishio (Shinkoiwa Clinic), Atsushi Hayama (Moriyama Rehabilitation Hospital), Toshio Shinoda (Kawakita General Hospital Dialysis Center), Takashi Kono (Mihama Narita Clinic), Takahiro Mochizuki (Kameda Medical Center), Yasuo Kimura (Shin-kashiwa Clinic), Noriyoshi Murotani (Chiba Social Insurance Hospital), Satoshi Yamaguchi (Asahi Hospital), Taichi Nakanishi (Kurihama Clinic), Kiyoshi Ozawa (Yokosuka Clinic), Takashi Nagaoka (Sagamihara Clinic), Takao Suga (Bousei Hiratsuka Clinic), Masakazu Suda (Suda Medical Clinic), Yoshikazu Goto (Saiyu Soka Hospital), Michio Kuwahara (Shuwa General Hospital Hemodialysis Clinic), Hiromi Shimoyama (Yuai Clinic), Kimihiko Matsuyama (Misato Kenwa Clinic), Kazue Ueki (Toho Hospital), Kyoko Ito (Heisei Hidaka Clinic), Katsuhiko Miyamoto (Seseragi Hospital), Takashi Ishizu (Tukuba Central Hospital), Shuichi Kikuchi (Ohba Renal Clinic), Masaki Kobayashi (Tokyo Medical University Ibaraki Medical Center), Mitsuyoshi Furuhashi (Maruyama Hospital), Masanori Wakabayashi (Bousei Dai-ichi Clinic), Kazuyoshi Nakamura (Fujidaiichi Clinic), Hirotake Kasuga (Kaikoukai Central Clinic), Itsuo Yokoyama (Nagoya Memorial Foundation Narumi Clinic), Chikao Yamazaki (Masuko Clinic SUBARU), Kijun Nagata (Sawada Hospital), Yasumasa Kawade (Suzuka Kidney Clinic), Toshiaki Kawanaka (Ishikiriseiki Hospital), Yoshihiro Tsujimoto (Inoue Hospital), Mikio Okamura (Ohno Memorial Hospital), Shigeki Okada (Okada Clinic), Senji Okuno (Kidney Center Shirasagi Clinic), Harumi Nagayama (Nagayama Hemodialysis Clinic), Shuji Okazaki (Nagayama Hospital), Yoshinori Tone (Fujii Clinic), Ibuki Yajima (Ibuki Clinic), Kouji Shibuya (Sumiyoshigawa Hospital), Kunihiko Yoshiya (Hara Genitourinary Hospital), Morihiro Kondou (Otowa Kinen Hospital), Satoru Yamazaki (Tojinkai Hospital), Ryoichi Miyazaki (Fujita Memorial Hospital), Katsuhiko Arimoto (Shigei Medical Research Hospital), Misaki Moriishi (Nakajima Tsuchiya Clinic), Takahito Nasu (Tokuyama Central Hospital), Seiichi Obayashi (Kinashi Obayashi Hospital), Yuzuru Sato (Sato Junkankika Naika), Takao Tanaka (Ohji Hospital), Hidetoshi Nakamura (Kokura Daiichi Hospital), Nobuhiko Koga (Shin-Koga Clinic), Harumichi Higashi (St. Mary’s Hospital), Kougi Yuu (Takahashi Naika Clinic), Asako Kitamura (Chikuho Social Insurance Hospital), Tomoji Matsumae (Murakami Memorial Hospital), Katsushige Abe (Jinikai Hospital), Masahiro Kawatomi (Kawatomi Internal Medicine Clinic), Motoko Tanaka (Akebono Clinic), Chisa Nogami (Kumamoto Urological Hospital), Etsuo Yoshidome (Ikeda Hospital), Shinyu Miyagi (Okinawa Daiichi Hospital), Satoshi Nakazato (Chibana Clinic), Yoshiki Shiohira (Tomishiro Central Hospital), and Kiyoyuki Tokuyama (Tokuyama Clinic).
