# Supplementary material for: The cardiothoracic ratio and all-cause and cardiovascular disease mortality in patients undergoing maintenance hemodialysis: results of the MBD-5D study
Source: Clin Exp Nephrol. 2017 May 15;21(5):797–806. doi: 10.1007/s10157-017-1380-2 (PMC5648748; doi:10.1007/s10157-017-1380-2)
Supplement: Supplementary file 1 — Supplementary material 1 (DOCX 143 KB) [file 10157_2017_1380_MOESM1_ESM.docx]

LEGEND

sFigure 2. Subgroup analysis stratified by the presence of diabetes mellitus (DM). Vertical line indicates the reference level. The adjusted HRs are shown with point estimates and 95% confidence intervals. HRs were adjusted by age, sex, BMI, smoking status, comorbidity of CVD, diabetes mellitus, dialysis duration, levels of iPTH, phosphorus, calcium, hemoglobin, albumin and C-reactive protein, Kt/V, dialysate calcium level, phosphate binder, vitamin D receptor activator, ACE inhibitor and beta blocker.

LEGEND

sFigure 3. Subgroup analysis stratified by age (<64 yr, >65 yr). Vertical line indicates the reference level. The adjusted HRs are shown with point estimates and 95% confidence intervals. HRs were adjusted by age, sex, BMI, smoking status, comorbidity of CVD, diabetes mellitus, dialysis duration, levels of iPTH, phosphorus, calcium, hemoglobin, albumin and C-reactive protein, Kt/V, dialysate calcium level, phosphate binder, vitamin D receptor activator, ACE inhibitor and beta blocker.
